# Extracytoplasmic function σ factors of the widely distributed group ECF41 contain a fused regulatory domain

**DOI:** 10.1002/mbo3.22

**Published:** 2012-06

**Authors:** Tina Wecke, Petra Halang, Anna Staroń, Yann S Dufour, Timothy J Donohue, Thorsten Mascher

**Affiliations:** 1 Department of Biology I, Ludwig-Maximilians-University MunichGermany; 2 Department of Bacteriology, University of WisconsinMadison, Wisconsin

**Keywords:** Anti-σ factor, ECF σ factor, signal transduction

## Abstract

Bacteria need signal transducing systems to respond to environmental changes. Next to one- and two-component systems, alternative σ factors of the extra-cytoplasmic function (ECF) protein family represent the third fundamental mechanism of bacterial signal transduction. A comprehensive classification of these proteins identified more than 40 phylogenetically distinct groups, most of which are not experimentally investigated. Here, we present the characterization of such a group with unique features, termed ECF41. Among analyzed bacterial genomes, ECF41 σ factors are widely distributed with about 400 proteins from 10 different phyla. They lack obvious anti-σ factors that typically control activity of other ECF σ factors, but their structural genes are often predicted to be cotranscribed with carboxymuconolactone decarboxylases, oxidoreductases, or epimerases based on genomic context conservation. We demonstrate for *Bacillus licheniformis* and *Rhodobacter sphaeroides* that the corresponding genes are preceded by a highly conserved promoter motif and are the only detectable targets of ECF41-dependent gene regulation. In contrast to other ECF σ factors, proteins of group ECF41 contain a large C-terminal extension, which is crucial for σ factor activity. Our data demonstrate that ECF41 σ factors are regulated by a novel mechanism based on the presence of a fused regulatory domain.

## Introduction

Bacteria populate complex habitats in which extracellular conditions can change very rapidly. In order to survive in such an environment, bacterial cells have to be able to sense and respond to these variations before cell damage actually occurs. Therefore, bacteria need signal transducing systems that enable them to sense these extracellular changes and respond by differential gene expression.

A common mechanism to control gene expression at the level of transcription initiation is the use of σ factors, which constitute an essential subunit of the RNA polymerase (RNAP) holoenzyme and determine the promoter specificity. In addition to the primary σ factor, which is responsible for general expression of most genes in exponentially growing cells, most bacteria contain one or more alternative σ factors. These proteins are activated only under certain conditions and control expression of a specific set of target genes by recognizing alternative promoter sequences (Helmann and Chamberlin 1988; Helmann 2010).

Most σ factors belong to the σ^70^ family based on their relation to the primary σ factor of *Escherichia coli*, σ^70^ (Gruber and Gross 2003; Paget and Helmann 2003). Based on sequence similarity, domain architecture and function, the proteins of the σ^70^ family can be divided into four groups. Group 1 comprises the essential primary σ factors, which contain four highly conserved domains (designated σ_1_ through σ_4_) (Gruber and Gross 2003). Group 2 σ factors are closely related to group 1 proteins, but are not essential for growth. The group 3 σ factors lack the σ_1_ domain and have functions in cellular processes such as sporulation, flagella biosynthesis, or heat shock response. The largest and most diverse group 4 contains the proteins of the ECF family, named after their function in response to extracellular stimuli (Lonetto et al. 1994; Helmann 2002; Butcher et al. 2008).

In contrast to other σ^70^ proteins, the ECF σ factors only contain two of the four conserved domains, σ_2_ and σ_4_, which are sufficient for promoter recognition and interaction with RNAP. The bipartite promoter recognized by ECF σ factors typically contains a highly conserved “AAC” signature in the –35 region and a “CGT” motif in the –10 region (Helmann 2002). In general, ECF σ factors autoregulate their own expression and are cotranscribed with a gene encoding an anti-σ factor that regulates the activity of the σ factor. In the absence of a stimulus, the anti-σ factor binds the ECF σ factor and keeps it inactive. Upon receiving the appropriate signal, the anti-σ factor often gets inactivated, thereby releasing and activating the σ factor (Helmann 2002; Butcher et al. 2008). The major principles of σ factor activation are based on either the regulated proteolysis of a membrane-anchored anti-σ factor as exemplified by RseA-σ^E^ of *E. coli* and RsiW-σ^W^ of *Bacillus subtilis* (Ades 2004; Heinrich and Wiegert 2009) or conformational changes of a soluble anti-σ factor, as has been described for RsrA-σ^R^ of *Streptomyces coelicolor* (Kang et al. 1999; Campbell et al. 2008). For yet other examples, such as *S. coelicolor* σ^E^ or EcfG-homologs in α-proteobacteria, two-component systems play a crucial role in regulating the activity of the ECF σ factors (Hong et al. 2002; Francez-Charlot et al. 2009).

A recent classification of the ECF σ factor protein family based on sequence similarity and genomic context conservation revealed a wide distribution and combinatorial complexity of ECF-dependent signal transduction. This study identified more than 40 phylogenetically distinct groups of ECF σ factors including major groups containing the *E. coli* σ^E^- and FecI-like proteins as well as cytoplasmic-sensing ECF σ factors. But in addition to these well-understood examples, a number of ECF groups were identified that have not yet been investigated experimentally (Staroń et al. 2009).

Here, we describe the characterization of one such uncharacterized group, ECF41. This group is widely distributed with about 400 proteins from 10 different phyla. Based on their genomic organization, the genes encoding these ECF41 σ factors are not transcriptionally linked to genes encoding proteins related to known anti-σ factors. Instead, they are located next to genes encoding carboxymuconolactone decarboxylases, oxidoreductases, or epimerases. To extract general features of ECF41-dependent gene regulation, we experimentally investigated ECF41 σ factors from two different organisms, *B. licheniformis* (Firmicutes) and *Rhodobacter sphaeroides* (α-proteobacteria). In both organisms, the ECF41 σ factor appears to control expression of a single transcript that is preceded by a highly conserved ECF41-specific promoter motif. A unique feature of ECF41 proteins is the presence of a large C-terminal extension containing a number of conserved signature motifs. We provide evidence that this C-terminal extension is involved in regulation of σ factor activity and we propose that it functions as a fused anti-σ factor-like domain.

## Materials and Methods

### Bioinformatics analysis

A total of 510 ECF41 proteins were extracted in October 2010 from the MiST2 database (Ulrich and Zhulin 2010) available at http://mistdb.com. False positives (unclassified ECF σ factors) and redundant proteins (proteins from more than one sequenced strain per species) were removed leaving 373 sequences for further analysis. Multiple sequence alignments were performed using ClustalW (Thompson et al. 1994), and phylogenetic trees were generated from gapless multiple sequence alignments using the Neighbor-Joining method of the Phylip (Felsenstein 1989) program Protdist, both implemented in the BioEdit program package (Hall 1999). Genomic context analysis was performed using the databases MicrobesOnline (Alm et al. 2005) at http://www.microbesonline.org and MiST2 (Ulrich and Zhulin 2010) available at http://mistdb.com/. Protein domain architecture was analyzed using the SMART database (Schultz et al. 1998; Letunic et al. 2006) available at http://smart.embl-heidelberg.de/.

Two hundred fifty base pairs region upstream of the genes encoding the ECF41 σ factors and the corresponding carboxymuconolactone decarboxylases, oxidoreductases, or epimerases (COE) were analyzed for putative promoter motifs either manually or with the help of MEME (Bailey and Elkan 1994), available at http://meme.nbcr.net/. Conservation of putative target promoters was illustrated using the WebLogo tool (Crooks et al. 2004) at http://weblogo.berkeley.edu. The promoter sequence of group ECF41 σ factors was used to screen the genomes of *R. sphaeroides* 2.4.1 and *B. licheniformis* DSM13 for putative target genes with the help of the virtual footprint algorithm (Münch et al. 2005), implemented into the Prodoric database (Münch et al. 2003) at http://www.prodoric.de/vfp/. As input pattern, the generated position weight matrix or the promoter consensus as IUPAC code was used.

### Bacterial strains and growth conditions

*Bacillus subtilis*, *B. licheniformis*, and *E. coli* were grown in LB medium at 37°C with aeration. *Rhodobacter sphaeroides* was grown aerobically in Sistrom's minimal medium (Sistrom 1960) at 30°C. All strains used in this study are listed in Table 1. The antibiotics spectinomycin (100 μg/mL), chloramphenicol (5 μg/mL), and erythromycin (1 μg/mL) plus lincomycin (25 μg/mL) for macrolide-lincosamide-streptogram (MLS) resistance were used for selection of *B. subtilis* and *B. licheniformis* mutants. Plasmid containing *E. coli* strains were grown with ampicillin (100 μg/mL) or kanamycin (50 μg/mL). *Rhodobacter sphaeroides* mutants were selected using tetracycline (1 μg/mL), spectinomycin (25 μg/mL), or kanamycin (25 μg/mL).

**Table 1:** Bacterial strains used in this study

Strain	Genotype or characteristic(s)	Source orreference
*E. coli* strains		
S17–1	C600::RP-4 2-(Tc::Mu)(Km::Tn*7*) *thi pro hsdR hsdM*^+^*recA*	Simon et al. 1983
DH5α	*recA1 endA1 gyrA96 thi hsdR17*(r_K_- m_K_+) *relA1 supE44 Φ*80Δ*lacZ*Δ*M15* Δ(*lacZYA-argF*)*U169*	Sambrook and Russell 2001
*B. subtilis* strains		
W168	Wild-type strain, *trpC2*	Laboratory stock
1A774	JH642 *rpoC*::(His_6_-tag) Spec^R^	BGSC (C. Moran)
TMB1099	1A774 pPH0401	This study
TMB1100	1A774 pPH0403	This study
TMB1101	1A774 pTW0412	This study
TMB695	W168 pPH0401	This study
TMB746	W168 pPH0403	This study
TMB666	W168 pTW0412	This study
TMB428	W168 *thrC*::pTW6302	This study
TMB455	TMB428 *amyE*::pTW901	This study
TMB451	W168 *amyE*::pTW901	This study
TMB456	TMB428 *amyE*::pTW902	This study
TMB858	W168 *amyE*::pPH901	This study
TMB574	TMB451 *thrC*::pTW6304	This study
TMB575	TMB451 *thrC*::pTW6305	This study
TMB577	TMB451 *thrC*::pTW6307	This study
TMB896	TMB858 *thrC*::pTW6304	This study
TMB897	TMB858 *thrC*::pTW6305	This study
TMB899	TMB858 *thrC*::pTW6307	This study
TMB623	TMB428 pHCMC04	This study
TMB696	TMB428 pPH0401	This study
TMB742	TMB428 pPH0403	This study
TMB741	TMB428 pPH0402	This study
TMB667	TMB428 pTW0412	This study
*B. licheniformis* strains		
DSM13	Wild-type strain	Laboratory stock
MW3	DSM13Δ*hsdR*1 Δ*hsdR*2	Waschkau et al. 2008
TMBli003	MW3 Δ*ydfG*	This study
TMBli006	MW3 Δ*ecf*41_Bli_	This study
*R. sphaeroides* strains		
2.4.1	Wild-type strain	Laboratory stock
TMR003	2.4.1 pTW0503	This study
TMR005	YSD418 pTW0501	This study
TMR006	YSD418 pTW0502	This study
TMR007	YSD418 pTW0503	This study
YSD418	2.4.1 P_RSP_0606_::pSUP202-*lacZ*	This study
YSD354	2.4.1 pIND4	This study
YSD239	2.4.1 ΔRSP_0606-*ecf*41_Rsp_ Ω::Spec^R^	This study
YSD331	YSD239 pIND4	This study
YSD333	2.4.1 pYSD161	This study
YSD434	YSD418 pIND4	This study

### DNA manipulations

Standard cloning techniques were applied (Sambrook and Russell 2001). All plasmids used in this study are listed in Table 2, oligonucleotides in Table S2. *Escherichia coli* strain S17–1 (Simon et al. 1983) was used for conjugational DNA transfer in *R. sphaeroides*. In brief, a 1:1 cell mixture of exponentially growing donor and recipient strains were harvested, washed, and resuspended in LB medium. The cell mixture was applied to a filter disc and incubated overnight on an LB plate at 30°C. The filter disc was transferred to Sistrom's minimal medium (Sistrom 1960) and incubated for 3 h at 30°C on a shaker, before the cells were plated on agar plates with selection. Conjugants were obtained after three to four days of incubation at 30°C.

**Table 2:** Vectors and plasmids used in this study

Name	Genotype or characteristic features^1^	Primers for cloning	Source or reference
Vectors			
pDG1663	*lacZ* fusion vector, integrates in *thrC*, MLS^R^		Guerout-Fleury et al. 1996
pHCMC04	Xylose-inducible expression vector, Cm^R^		Nguyen et al. 2005
pIND4	IPTG-inducible expression vector, Kn^R^		Ind et al. 2009
pSUP202	Mobilizable vector, Ap^R^, Cm^R^, Tc^R^		Simon et al. 1983
pSWEET	Xylose-inducible expression vector, integrates in *amyE*, Cm^R^		Bhavsar et al. 2001
pMAD	Shuttle vector for construction of makerless deletion mutans, MLS^R^		Arnaud et al. 2004
pHP45Ω	Source of Ω::Spec^R^ cassette		Prentki and Krisch 1984
pGEM-T	Cloning vector		Promega Corp.
Plasmids			
pTW101	pMAD *ecf*41_Bli_ up/do	779/780, 781/782	This study
pTW102	pMAD *ydfG* up/do	783/784, 785/786	This study
pTW6302	pDG1663 P*_ydfG_*_(-146–54)_-*lacZ*	712/713	This study
pTW6304	pDG1663 P*_nhaX_*_(-355–40)_-*lacZ*	1130/1131	This study
pTW6305	pDG1663 P*_ybpE_*_(-111–63)_-*lacZ*	1136/1137	This study
pTW6307	pDG1663 P*_uvrX_*_(-173–54)_-*lacZ*	1132/1133	This study
pTW901	pSWEET *ecf*41_Bli_	699/669	This study
pTW902	pSWEET *ecf*41_Bli_*-ydfG*	699/705	This study
pPH901	pSWEET *ecf*41_Bliaa1–204_	699/1576	This study
pPH0401	pHCMC04 *ecf*41_Bli_-FLAG	1416/1294	This study
pPH0403	pHCMC04 *ecf*41_Bli aa1–204_-FLAG	1416/1469	This study
pPH0402	pHCMC04 *ecf*41_Bli aa1–192_-FLAG	1416/1468	This study
pTW0412	pHCMC04 *ecf*41_Bli aa1–167_-FLAG	1416/1411	This study
pSUP202-*lacZ*	pSUP202 with promoter-less *lacZ* gene	199/200	This study
pSUP202-P_RSP_0606_-*lacZ*	P_RSP_0606_ fused to *lacZ* gene	109/219	This study
pYSD122	pSUP202 with the Ω::Spec^R^ cassette and genomic regions flanking RSP_0606-*ecf*41_Rsp_	109/110/125/126	This study
pYSD161	pIND4 *ecf*41_Rsp_	185/186	This study
pTW0501	pIND4 *ecf*41_Rsp_	1881/1603	This study
pTW0502	pIND4 *ecf*41_Rsp aa1–169_	1881/1604	This study
pTW0503	pIND4 *ecf*41_Rsp aa1–206_	1881/1605	This study

1 Resistance cassettes: MLS^R^, macrolide-lincosamide-streptogram; Cm^R^, chloramphenicol; Kn^R^, kanamycin; Ap^R^, ampicillin; Tc^R^, tetracycline; Spec^R^, spectinomycin.

### Construction of markerless *ecf*41_Bli_ and *ydfG* deletion mutants in *B. licheniformis*

Markerless *B. licheniformis* Δ*ecf*41_Bli_ and Δ*ydfG* mutants were constructed using the vector pMAD (Arnaud et al. 2004). Seven hundred base pairs fragments up- and downstream of *ecf*41_Bli_ and *ydfG* were amplified by PCR using the oligonucleotides listed in Table S2, introducing extensions at the 3′ end of the up fragments that are complementary to the 5′ end of the down fragments. These regions were used to fuse the fragments in a second joining PCR. The resulting products were then cloned into pMAD using BamHI and EcoRI generating pTW101 and pTW102. The plasmids were introduced into *B. licheniformis* MW3 as described (Waschkau et al. 2008). Generation of markerless deletion mutants basically followed the established procedure (Arnaud et al. 2004). In brief, transformants were incubated at 30°C with MLS selection on LB agar plates supplemented with X-Gal. Blue colonies were picked and incubated for 6–8 h at 42°C in LB medium with MLS selection, resulting in the integration of the plasmid into the chromosome. Again, blue colonies were picked from LB X-Gal plates and incubated for 6 h at 30°C in LB medium without selection. Subsequently, the liquid culture was shifted to 42°C for 3 h, and the cells were then plated on LB X-Gal plates, this time without selective pressure. White colonies that had lost the plasmid were picked and deletion of *ecf*41_Bli_ or *ydfG* was checked by PCR.

### Construction of an RSP_0606-*ecf*41_Rsp_ deletion mutant in *R. sphaeroides*

RSP_0606-*ecf*41_Rsp_ with 1.3 kb flanking regions on both sides was amplified from chromosomal DNA of *R. sphaeroides* using oligonucleotides 109 and 110 (Table S2) and ligated into the vector pGEM-T (Promega, Madison). To replace RSP_0606-*ecf*41_Rsp_ with a resistance cassette, the regions flanking the genes and the plasmid were amplified using internal oligonucleotides (125, 126) and ligated to the Ω fragment derived from pHP45Ω (Prentki and Krisch 1984), conferring spectinomycin resistance. The resulting construct was amplified using oligonucleotides 109 and 110, cloned into the suicide vector pSUP202 (which contains a tetracycline resistance marker) digested with ScaI to make pYSD122. The plasmid pYSD122 was than conjugated into *R. sphaeroides* 2.4.1. Double recombinants corresponding to the deletion mutants were selected for spectinomycin resistance and sensitivity to tetracycline. Plasmid constructs were verified by sequencing, and the deletion in the *R. sphaeroides* genome was verified by PCR.

### Measurement of promoter activity by β-galactosidase assays

Because of the lack of genetic tools for *B. licheniformis*, we developed a heterologous expression system in *B. subtilis*, an organism lacking an ECF41 σ factor. A DNA fragment from *B. licheniformis* containing the intergenic region between *ydfG* and *ecf*41_Bli_ was fused to a promoter-less *lacZ* gene using the vector pDG1663 and integrated into the *thrC* locus of *B. subtilis*. In addition, we fused a FLAG-tag to the N-terminus of Ecf41_Bli_ and its truncated versions and expressed the protein from the xylose-inducible promoter of the shuttle vector pHCMC04, allowing determination of P*_ydfG_* activity by β-galactosidase assays in response to Ecf41_Bli_ expression.The resulting *B. subtilis* strains were inoculated from fresh overnight cultures and grown in LB medium at 37°C with aeration until they reached an OD_600_ of ∼0.4. The cultures were split and 0.5% xylose was added to one sample to induce expression of Ecf41_Bli_ from the inducible promoter. After incubation for 1 h at 37°C, 2 mL of each sample were harvested and the cell pellets frozen at –20°C. The pellets were resuspended in 1 mL working buffer and assayed for β-galactosidase activity with normalization to cell density (Miller 1972).

A DNA fragment containing the upstream region of RSP_0606 was amplified and cloned into the suicide vector pSUP202 carrying a promoter-less *lacZ* gene. The resulting plasmid was conjugated into *R. sphaeroides* and integrated into the chromosome by single crossing over, thereby bringing the expression of β-galactosidase under control of P_RSP_0606_. Full-length and truncated *ecf*41_Rsp_ was amplified and cloned into the overexpression vector pIND4, thereby bringing its expression under control of an IPTG-inducible promoter. The resulting *R. sphaeroides* strains were grown aerobically in Sistrom's minimal medium (Sistrom 1960) to an OD_600_ of ∼0.3. The cultures were split and expression of Ecf41_Rsp_ was induced in one sample by adding 100 μM IPTG. After 3 h, the cells were harvested and β-galactosidase activity was measured as described (Miller 1972).

### Preparation of total RNA

*Bacillus licheniformis* MW3 (wt) and TMBli003 (Δ*ecf*41_Bli_) were grown aerobically in LB medium at 37°C. Every 2 h, 30 mL samples were taken and mixed with cold killing buffer (20 mM Tris-HCl, pH 7.0, 0.5 mM MgCl_2_, 20 mM NaN_3_), harvested by centrifugation, and frozen in liquid nitrogen, before the pellets were stored at –80°C. The cells were resuspended in 200 μL killing buffer, immediately transferred to a precooled Teflon vessel and disrupted with a Micro-Dismembrator U (Sartorius) for 3 min at 2000 rpm. The resulting cell powder was resuspended in 3 mL prewarmed lysis solution (4 M guanidine-thiocyanate, 25 mM sodium acetate, pH 5.2, 0.5% *N*-lauroyl sarcosinate) and total RNA was extracted twice with acid phenol (phenol/chloroform/isoamylalcohol 25/24/1, pH 4.5–5) and once with chloroform (chloroform/isoamylalcohol 24/1) followed by isopropanol precipitation. Contaminating DNA was removed using the Baseline-ZERO DNAse (Epicentre Biotechnologies, Madison) according to the manufacturer's protocol. RNA was quantified with a NanoDrop 1000 Spectrophoto-meter (Thermo Scientific, Schwerte) and used for 5′RACE and Northern Blot analysis.

*Rhodobacter sphaeroides* YSD354 (pIND4) and YSD333 (pYSD161) were grown aerobically in Sistrom's minimal medium (Sistrom 1960) containing 25 μg/mL kanamycin at 30°C. At OD_600_ of ∼0.3, expression of Ecf41_Rsp_ was induced by adding 100 μM IPTG. After 3 h, 44 mL of culture was mixed with 6 mL stop solution (5% acid phenol in ethanol) and harvested by centrifugation. The pellets were frozen in an ethanol/dry ice bath and stored at –80°C. Cells were resuspended in 2 mL lysis solution (2% SDS, 16 mM EDTA) and incubated at 65°C for 5 min. RNA was extracted three times with acid phenol prewarmed to 65°C followed by chloroform extraction and isopropanol precipitation. To remove contaminating DNA, the RNA was incubated with two units RQ1 DNase (Promega, Madison) in the presence of 80 units RNasin Plus RNase Inhibitor (Promega, Madison) for 30 min at 37°C. The RNA was finally purified with the RNeasy Mini Kit (Qiagen, Hilden) and used for DNA Microarray analysis and 5′RACE.

### Probe preparation and Northern Blot analysis

An ∼500 bp internal fragment of *ydfG* was amplified by PCR with oligonucleotides listed in Table S2. A digoxigenin (DIG)-UTP-labeled RNA probe was synthesized by in vitro transcription using the DIG RNA Labeling Mix (Roche, Mannheim) and T7 RNA polymerase (Roche, Mannheim) according to the manufacturer's protocol.

For Northern Blot analysis, 10 μg of total RNA was separated under denaturing conditions on a 1% formaldehyde agarose gel and transferred to a positively charged nylon membrane (Roche, Mannheim) in a downward transfer using 20× SSC (3 M NaCl, 0.3 M sodium citrate, pH 7.0) as transfer buffer. The RNA was crosslinked by exposing the membrane to UV light. The blot was prehybridized at 68°C for 1 h with hybridization solution (5× SSC, 50% formamide, 2% Blocking Reagent (Roche, Mannheim), 0.1% *N*-lauroyl sarcosinate, and 0.02% SDS). Hybridization was carried out overnight at 68°C in the same solution with 1 μg DIG-labeled RNA probe. The membrane was washed twice for 5 min at room temperature (2× SSC, 0.1% SDS) and three times for 15 min at 68°C (0.1× SSC, 0.1% SDS). The signal was detected with an antidigoxigenin antibody conjugated to alkaline phosphatase (Roche, Mannheim) and CDP-*Star* (Roche, Mannheim) according to the manufacturer's instructions. The signals were visualized using a LumiImager (Peqlab, Erlangen).

### DNA microarray analysis

RNA samples from three independent cultivations were used for cDNA synthesis and DNA microarray hybridization. A total of 10 μg of total RNA was mixed with 3 μg random hexamers and denatured at 70°C for 10 min before the temperature was decreased in six cycles (1 min each) by 10°C/cycle to 10°C to optimize annealing of the hexamers. cDNA was synthesized using SuperScript II Reverse Transcriptase (Invitrogen, Karlsruhe) according to the manufacturer's instruction. Temperature was increased from 20°C to 42°C in 22 cycles of 3 min with 1°C increment followed by incubation at 42°C for 1 h and inactivation at 70°C for 10 min. Remaining RNA was removed by alkaline hydrolysis and cDNA was purified using the PCR Purification Kit (Qiagen, Hilden). A total of 3.2 μg cDNA was fragmented with 0.25 units RQ1 DNase (Promega, Madison) for 10 min at 37°C followed by inactivation for 10 min at 98°C. cDNA was labeled using the BioArray Terminal Labeling Kit with Biotin-ddUTP for DNA Probe Array Assays (Enzo, Farmingdale) according to the manufacturer's protocol. Labeled cDNA samples (3 μg/array) were hybridized to Affymetrix (Santa Clara, CA) custom arrays (Pappas et al. 2004) according to the manufacturer's directions. Processing, normalization, and statistical analysis of the array data were performed in the R Statistical Software environment (http://www.r-project.org/). Data were normalized using the *affyPLM* package with default settings (Bolstad 2004). Differentially expressed genes were detected using the *limma* package with a false discovery rate set at 0.05 (Smyth 2005).

### Determination of transcriptional start sites by 5′-RACE

The 5′ ends of *ydfG* and RSP_0606 mRNAs were identified by rapid amplification of cDNA ends (RACE). A total of 15 μg of total RNA was incubated with 25 units tobacco acid pyrophosphatase (TAP, Epicentre Biotechnologies, Madison) in the delivered buffer at 37°C for 60 min in the presence of 40 units Super RNaseIn RNAse inhibitor (Ambion, Austin). As a control, 15 μg RNA was incubated under the same conditions, but without TAP. The reactions were phenol/chloroform extracted and ethanol precipitated. After dissolving the pellets in water, the RNA was mixed with 500 pmol RACE adapter (5′-GAUAUGCGCGAAUUCCUGUAGAACGAACACUAGAAGAAA-3′) and denatured at 95°C for 5 min. Ligation of the adapter was carried out at 17°C overnight with 100 units T4 RNA ligase (Epicentre Biotechnologies, Madison) in the presence of 80 units Super RNaseIn RNAse inhibitor (Ambion, Austin). Again, the reactions were phenol/chloroform extracted, ethanol precipitated, and the pellets were resuspended in water. One microgram RNA was used for reverse transcription with gene-specific primers (GSP1, Table S2) and the iScript Select cDNA Synthesis Kit (Bio-Rad, München) according to the manufacturer's protocol. The cDNA was then amplified with nested primers and a primer complementary to the RACE adapter sequence (GSP2 and 679, Table S2) and the transcription start sites were identified by sequencing.

### Western Blot analysis

*Bacillus subtilis* strains containing overexpression plasmids were grown in LB medium at 37°C to an OD_600_ of ∼0.4. Expression of Ecf41_Bli_-FLAG and its variants was induced by adding 0.5% xylose. After 1 h, 15 mL of each culture was harvested. The pellets were resuspended in ZAP buffer (10 mM Tris, pH 7.4, 200 mM NaCl), cells were lysed by sonication, and cell debris was removed by centrifugation. A total of 20 μg of the cleared lysate was separated by SDS-PAGE and transferred to a Polyvinylidene difluoride (PVDF) membrane using a Mini Trans-Blot Electrophoretic Transfer Cell (Bio-Rad, München) according to the manufacturer's instructions. The membrane was then incubated overnight at 4°C with blotto (2.5% skim milk in TBS [50 mM Tris, pH 7.6, 150 mM NaCl]) to prevent nonspecific binding. Then, the membrane was incubated with the primary antibody (anti-FLAG [Sigma, München] diluted 1:2000 in blotto) at room temperature for 1 h followed by four 10 min washing steps with blotto. Then the blot was incubated for 1 h with the secondary antibody (anti-rabbit IgG HRP conjugate [Promega] diluted 1:2000 in blotto). After four washing steps with blotto, the membrane was washed with TBS before the signals were detected with a LumiImager (Peqlab, Erlangen) using AceGlow (Peqlab, Erlangen) as chemiluminescence substrate.

### RNAP pull-down assays

Different versions of Ecf41_Bli_-FLAG under control of a xylose-inducible promoter were introduced into *B. subtilis* 1A774, which contains a His_6_-tag fused to the β′ subunit of the RNAP, to form strains TMB1099 (wt Ecf41_Bli_), TMB1100 (Ecf41_Bli_ 204), and TMB1101 (Ecf41_Bli_ 167). As controls, the same constructs were transformed into *B. subtilis* W168 resulting in TMB695, TMB746, and TMB666. The RNA pull-down assays were performed as described previously (MacLellan et al. 2008). In brief, 100 mL LB medium supplemented with 5 μg/mL chloramphenicol was inoculated from fresh overnight cultures and grown till OD_600_ ∼0.4. Cultures were induced with 0.5% xylose for 1 h and cells were harvested by centrifugation. The pellets were resuspended in phosphate buffer (50 mM phosphate buffer, pH 7.6, 100 mM NaCl, 0.1 mM PMSF, 5 mM imidazole) and cells were lysed by sonication. The cleared lysate was loaded on a column containing 0.5 mL Ni-NTA metal affinity beads. The beads were washed with each 10 column volumes of the above-mentioned phosphate buffer containing 5, 10, and 20 mM imidazole. Elution was carried out using 0.5 mL phosphate buffer with increasing imidazole concentration (50, 100, 250, and 500 mM). Samples of the cleared lysate, washing steps, and elution fractions were run in duplicates on 10% and 12% SDS-PAGE gels and checked for presence of RNAP (coomassie staining) and Ecf41_Bli_-FLAG (Western Blot using anti-FLAG anti-bodies). For quantitative analysis, 5 μg of lysate as well as 5 and 10 μg of the 100 mM imidazole elution fractions were used and analyzed as mentioned above.

## Results

### In silico analysis of group ECF41 σ factors

#### Phylogenetic distribution

The initial analysis of group ECF41 (Staroń et al. 2009) was based on a dataset generated in 2008 containing 115 ECF41 protein sequences from five different phyla. To account for the huge increase in bacterial genomes sequenced within the last three years, we reanalyzed group ECF41 based on 373 ECF41 σ factors extracted from the Microbial Signal Transduction Database (MiST2) (Ulrich and Zhulin 2010) (Table S1).

Group ECF41 shows a wide taxonomic distribution and proteins of this group can be found in 10 different phyla, but predominantly in the Actinobacteria and Proteobacteria (Table 3). In addition, ECF41 σ factors can also be found in the phyla Firmicutes, Chloroflexi, Acidobacteria, Bacteriodetes, Cyanobacteria, Spirochaetes, Verrucomicrobia, and Gemmatimonadetes.

**Table 3:** Phylogenetic distribution of ECF41 σ factors

Phyla	ECF41 proteins per phylum	Percentage of ECF41 proteins	Species with ECF41 protein	Percentage of sequenced species	Sequenced genomes/species^1^
Actinobacteria	252	68	60	51	181/118
Proteobacteria	84	23	66	15	705/414
Firmicutes	15	4.0	10	2.5	404/182
Chloroflexi	11	2.9	3	60	15/5
Acidobacteria	4	1.1	4	67	6/6
Bacteriodetes	2	0.5	2	4.4	53/45
Cyanobacteria	2	0.5	2	6.3	44/32
Spirochaetes	1	0.3	1	5.6	23/18
Verrucomicrobia	1	0.3	1	25	4/4
Gemmatimonadetes	1	0.3	1	100	1/1

1 Numbers of sequenced genomes and species of each phylum were extracted from the MiST2 database (Ulrich and Zhulin 2010) in October 2010.

The 373 proteins of group ECF41 derive from 150 different species. Therefore, these organisms often encode more than one copy of the ECF41 gene in the genome (Tables 3 and S1). Especially within the Actinobacteria, multiple copies are very common. Only 14 of the 60 ECF41-containing actinobacterial species harbor just one copy of this σ factor, while the genomes of the remaining 46 contain several copies. Especially the genus *Streptomyces* contains large numbers of ECF41 σ factors with at least four copies per genome, which may reflect the complex lifestyle of these bacteria (Flärdh and Buttner 2009). The ECF41 copy number correlates well with the genome size and the overall abundance of signal trans-ducing systems. For example, the genome of *S. coelicolor* encodes as many as 45 ECF σ factors (Bentley et al. 2002), 13 of which belong to group ECF41. A high abundance of ECF41 genes can also be found in the phylum Chloroflexi (11 ECF41 σ factors/3 genomes), whereas most of the Proteobacteria (84 ECF41 σ factors/66 genomes) and Firmicutes (15 ECF41 σ factors/10 genomes) harbor only one to two ECF41 σ factors per genome.

**Figure 1:**
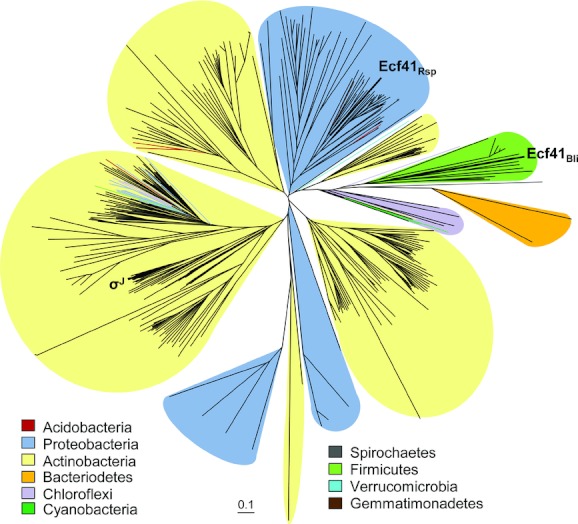
Phylogenetic tree of ECF41 σ factors. The phylogenetic tree is based on a gapless multiple sequence alignment of 373 ECF41 protein sequences constructed using ClustalW (Thompson et al. 1994). The resulting phylogenetic tree was calculated using the neighbor-joining method of the Phylip (Felsenstein 1989) program Protdist implemented in the BioEdit Sequence Alignment Editor (Hall 1999). Assignment to bacterial phyla is indicated by a color code. Ecf41_Rsp_ of *Rhodobacter sphaeroides*, Ecf41_Bli_ of *Bacillus licheniformis*, and σ^J^ of *Mycobacterium tuberculosis* are highlighted.

We constructed an unrooted phylogenetic tree based on a gapless multiple sequence alignment of all 373 ECF41 σ factors using the neighbor-joining method implemented in the Phylip program Protdist (Felsenstein 1989) provided by the BioEdit Sequence Alignment Editor (Hall 1999). In general, the terminal nodes representing sequences of ECF41 σ factors cluster according to the phylum (Fig. 1). The two phyla containing the highest number of sequences (Actinobacteria and Proteobacteria) are divided into five and three different branches, respectively. One cluster within one actinobacterial branch is rather diverse and includes ECF σ factors from Proteobacteria, Chloroflexi, Firmicutes, and Acidobacteria. The remaining ECF41 σ factors from Firmicutes as well as Chloroflexi form single branches. The ECF41 proteins from Bacteriodetes and Cyanobacteria also cluster together, whereas the proteins from Acidobacteria, Spirochaetes, Verrucomicrobia, and Gemmatimonadetes cluster within or between actinobacterial and proteobacterial branches (Fig. 1).

#### Genomic context conservation

**Figure 2:**
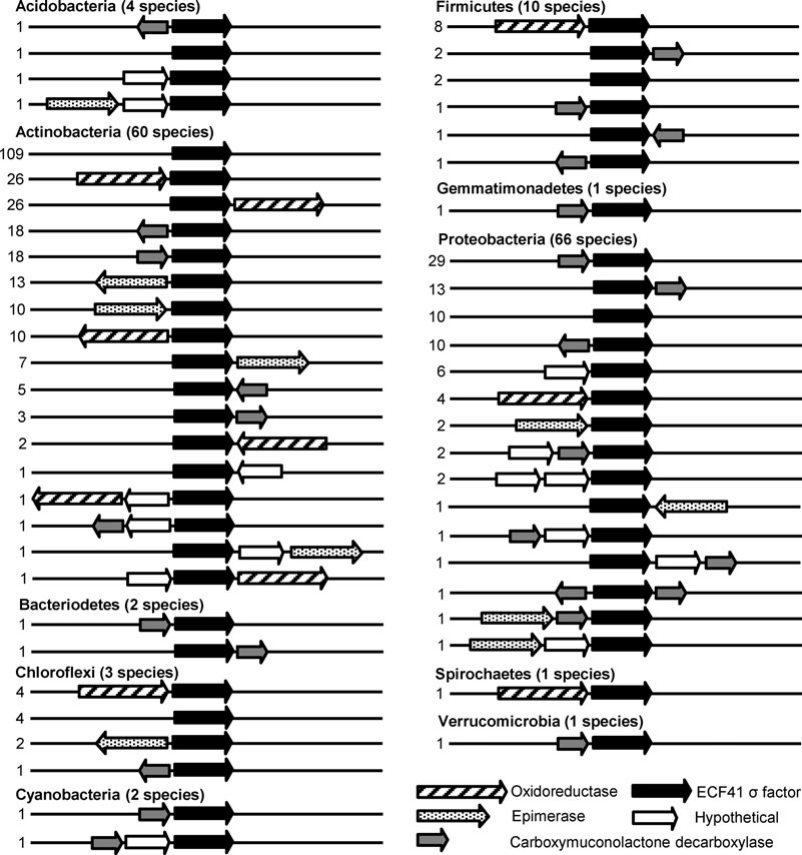
Genomic context conservation of ECF41 σ factors. ECF σ factors are shown by black, carboxymuconolactone decarboxylases by gray, oxidoreductases by striped and epimerases by dotted arrows. Genes encoding hypothetical proteins, that either contain the conserved ECF41-dependent promoter motif (Fig. 4) or are located between the ECF41 σ factor and the COE, are displayed in white. The genomic context is represented according to the phylum with the number of species in parentheses. The number in front of each context indicates how often this combination of genes occurs within the designated phylum.

In contrast to most ECF σ factors studied to date (Butcher et al. 2008), no gene encoding an obvious anti-σ factor can be found in direct vicinity of the genes encoding the ECF41 σ factors. Instead, they are genomically associated with genes encoding carboxymuconolactone decarboxylases, oxido-reductases, or epimerases (collectively referred to as “COE” from here on) (Fig. 2). While this genomic context is highly conserved, the order and orientation of the associated genes is diverse. In almost 50% of the cases, both genes are orientated in the same direction and could potentially be transcribed as an operon. In less than 20%, the genes are orientated divergently. The remaining ∼30% of ECF41 σ factors do not cluster with genes encoding COEs (Table 4). Such “orphans” are especially abundant in actinobacterial species (Fig. 2), which often contain multiple copies of ECF41 genes in the genome (Table S1). Here, at least one copy of the ECF41 genes shows the conserved genomic context.

**Table 4:** Genomic context and promoter occurrence

Genomic context^1^	Number	P_ECF_^2^	P_COE_^2^
>ECF>	126 (34%)	38	n.a.
>COE> >ECF>	107 (29%)	–	97
>ECF> >COE>	53 (14%)	14	24
>ECF> <COE<	9 (2%)	3	4
<ECF< >COE>	55 (15%)	36	41
Ungrouped	23 (6%)	n.a.	n.a.

1 The arrows indicate the organization of the genes. ECF, gene encoding an ECF41 σ factor; COE, gene encoding a carboxymuconolactone decarboxylase, oxidoreductase, or epimerase; ungrouped, genomic context differs from the above-mentioned groups and contains genes encoding hypothetical proteins of unknown function.

2 “-”, no promoter occurs upstream of the gene; n.a., the corresponding gene is not present or was omitted from analysis in case of ungrouped genomic context.

**Figure 4:**
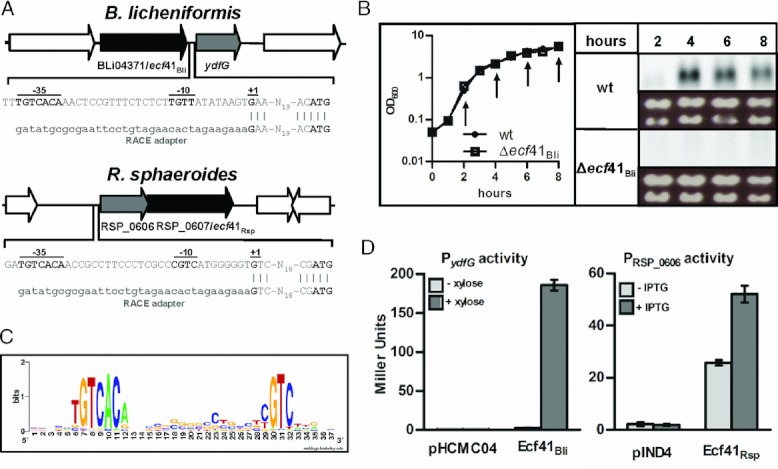
Targets of ECF41-dependent signal transduction. (A) Genomic context organization and target promoter sequence of the ECF41 σ factors from *B. licheniformis* and *R. sphaeroides*. Genes encoding the ECF41 σ factor (black) and the carboxymuconolactone decarboxylase (gray) as well as the promoter sequences are shown. Flanking genes not belonging to the ECF41 loci are shown in white. The –35 and –10 region, the transcriptional start site +1, and the ATG start codon are highlighted in bold. The RACE adapter sequence is indicated by lower case letters. (B) Northern Blot analysis of Ecf41_Bli_-dependent *ydfG* expression in *B. licheniformis*. *Bacillus licheniformis* MW3 (wt) and TMBli003 (Δ*ecf*41_Bli_) were grown aerobically in LB medium. At the time points indicated by arrows, samples of both strains were harvested and total RNA was prepared. A total of 10 μg total RNA was separated on a 1% formaldehyde gel and transferred to a nylon membrane followed by hybridization and detection with a DIG-labeled *ydfG*-specific probe. Ribosomal RNA is shown to ensure equal amounts of RNA in each lane. (C) Weblogo of ECF41-dependent target promoters. The weblogo was generated using the WebLogo tool (Crooks et al. 2004) available at http://weblogo.berkeley.edu. The weblogo graphically represents a position weight matrix and illustrates the degree of sequence conservation for each nucleotide. The matrix is based on 285 putative promoter sequences identified upstream of genes encoding ECF41 σ factors and COEs. (D) ECF41-dependent target promoter activation. *Bacillus subtilis* strains TMB696 (Ecf41_Bli_) and TMB623 (pHCMC04) were grown in LB medium to OD_600_ ∼0.4 and split into two samples. In one sample, expression of Ecf41_Bli_ was induced by addition of 0.5% xylose and cells were harvested after 1 h. *Rhodobacter sphaeroides* strains TMR005 (Ecf41_Rsp_) and YSD434 (pIND4) were grown in Sistrom's minimal medium to OD_600_ ∼0.3 and split into two samples. In one sample, expression of Ecf41_Rsp_ was induced by 100 μM IPTG. After 3 h, the cells were harvested. P*_ydfG_* and P_RSP_0606_ activities were measured by β-galactosidase assays with normalization to cell density.

*Carboxymuconolactone decarboxylases*. Commonly, proteins of the carboxymuconolactone decarboxylase family (PF02627) can be divided into two main groups: the γ-carboxymuconolactone decarboxylases and the AhpD-like alkylhydroperoxidases (Ito et al. 2006). The γ-carboxymuconolactone decarboxylases are involved in the degradation of aromatic compounds. They catalyze the decarboxylation of γ-carboxymuconolactone to β-ketoadipate enol-lactone in the protocatechuate branch of the β-ketoadipate pathway (Eulberg et al. 1998). The best investigated example of the second group is the alkylhydroperoxidase AhpD of *Mycobacterium tuberculosis*. This protein contains a CxxC motif critical for catalytic activity and is part of the antioxidant defense system of this organism (Hillas et al. 2000; Koshkin et al. 2003). In the archaeon *Methanosarcina acetivorans*, it was shown that the product of gene MA3736 encodes an uncharacterized carboxymuconolactone decarboxylase homolog with disulfide reductase activity dependent on a CxxC motif (Lessner and Ferry 2007). It was suggested to play a role in the oxidative stress response of this organism. All carboxymuconolactone decarboxylases genomically linked to ECF41 σ factors contain a conserved CxxC motif, suggesting a role of this group in the defense against oxidative stress.

*Oxidoreductases*. The reactions catalyzed by oxido-reductases can be very diverse, but are always characterized by the transfer of electrons from one molecule to another, often using NAD(P)H or FAD as cofactors. Since oxidoreductases can use a variety of different molecules as electron donor or acceptor, it is difficult to assign a specific function to these enzymes. In case of genes next to ECF41 σ factors, they were classified as oxidoreductase if their product carried at least one of the following Pfam domains: Oxidored_FMN, Flavodoxin_2, Pyr_redox/_2, FAD_binding_2/3/4, Amino_oxidase, Pyridox_oxidase, or FMN_red.

*Epimerases*. The third group contained either an NmrA (PF05368) or an Epimerase (PF01370) domain. NmrA is a negative transcriptional regulator of AreA and involved in nitrogen metabolite repression in different fungi. The crystal structure of NmrA revealed a Rossmann-fold and similarity to members of the short-chain dehydrogenase/reductase family (Stammers et al. 2001), which generally deploy nucleotide–sugar substrates for chemical conversions. The Rossmann-fold is typical for two-domain redox enzymes that use NAD^+^ as cofactor. The UDP-galactose 4-epimerase is the best understood example of this family and catalyzes the conversion of UDP-galactose to UDP-glucose (Allard et al. 2001).

*Miscellaneous*. In some cases, genes encoding other than the above-mentioned proteins can also be linked to ECF41 σ factors. These neighboring genes were included in Figure 2 if they (1) carry the typical promoter sequence (see below and Fig. 4), or (2) are located between the ECF41- and the COE-encoding genes. Most of these other genes encode hypothetical proteins of unknown function. Four of these hypothetical proteins (Table S1) contain the conserved β-barrel domain of the cupin superfamily, which members often function as dioxygenases in bacteria (Dunwell et al. 2004). The C-terminal domain of the cytoplasmic anti-σ factor ChrR from *R. sphaeroides* σ^E^ also adopts such a cupin fold (Campbell et al. 2007). In all four cases, the genes encoding these cupin fold proteins are in the same orientation than the ECF σ factor and presumably form an operon.

#### ECF41 proteins contain a large C-terminal extension

**Figure 3:**
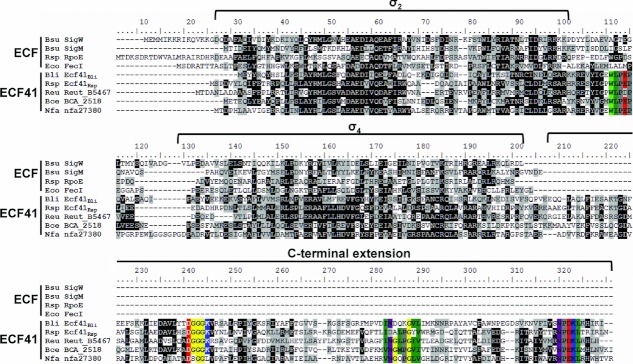
Characteristic features of group ECF41 proteins and comparison with classical ECF σ factors. The multiple sequence alignment of selected ECF σ factors was constructed using ClustalW (Thompson et al. 1994). Identical amino acids at the same position are shaded in black, similar amino acids in gray. The σ_2_ and σ_4_ domains and the C-terminal extension are marked. Conserved motives of ECF41 proteins are defined by the complete multiple sequence alignment of group ECF41 (Fig. S1) and are highlighted. Abbreviations: Bce, *Bacillus cereus*; Bli, *B. licheniformis*; Bsu, *B. subtilis*; Eco, *Escherichia coli*; Nfa, *Nocardia farcinica*; Rsp, *Rhodobacter sphaeroides*; Reu, *Ralstonia eutropha*.

Group 4 alternative σ factors contain the smallest proteins of the σ^70^ family, in which only regions σ_2_ and σ_4_ are present and sufficient for promoter recognition and RNAP interaction. An alignment of classical ECF σ factors from different organisms and proteins of group ECF41 revealed a large C-terminal extension of about 100 amino acids only present in ECF41 σ factors (Fig. 3). Based on an alignment of all ECF41 proteins (Fig. S1), we identified three conserved motifs within this extension (Fig. 3). Another characteristic feature of the ECF41 proteins is a highly conserved WLPEP motif in the linker region between σ_2_ and σ_4_, which usually does not show much sequence conservation in other ECF σ factors.

By analogy to other group 4 σ factors, we expect activity of the ECF41 proteins to be regulated in response to some unknown signal. Based on the observations regarding the genomic context and domain architecture of ECF41 σ factors, we propose three hypotheses to explain their regulation: (1) the COE genes could be targets of ECF41-dependent regulation, (2) the COEs could be part of the signal transducing mechanism and function as an anti-σ factor, or (3) the C-terminal extension could be involved in controlling σ factor activity.

To address these hypotheses directly and generalize our findings, we experimentally investigated ECF41 σ factors from two different organisms: BLi04371 of *B. licheniformis* and RSP_0607 of *R. sphaeroides*. We named the genes encoding the ECF41 σ factors to *ecf*41_Bli_ and *ecf*41_Rsp_ and used these terms for the following analysis. The genomic neighborhood including the genes encoding the carboxymuconolactone decarboxylases YdfG and RSP_0606 is shown in Figure 4A.

### Targets of ECF41 σ factors

#### COE-encoding genes represent targets of ECF41-dependent signal transduction

We first investigated if the COE-encoding genes next to the ECF41 σ factors are targets of the ECF41-dependent signal transduction. Therefore, we monitored expression of *ydfG* at different growth phases in a *B. licheniformis* wild-type and an isogenic Δ*ecf*41_Bli_ deletion strain. Both strains show no difference in growth behavior (Fig. 4B), indicating that Ecf41_Bli_ is not required under standard laboratory conditions. At designated time points, samples of both strains were taken, total RNA was prepared, and Northern Blot analysis was performed using a *ydfG*-specific probe. At the transition from the exponential to the stationary growth phase, an ∼0.5 kb transcript appears in the wild-type strain in agreement with a monocistronic expression of *ydfG* (Fig. 4B). No *ydfG* transcript is visible in the Δ*ecf*41_Bli_ deletion mutant, demonstrating that detectable expression of *ydfG* is completely Ecf41_Bli_-dependent under the conditions tested.

We also examined the transcriptome upon overexpression of Ecf41_Rsp_ in *R. sphaeroides* by DNA microarrays to test if a similar result can be obtained in another organism and to possibly identify additional target genes of ECF41 σ factors. Strains containing either an Ecf41_Rsp_ overexpression plasmid (YSD333) or the empty vector (YSD354) were grown aerobically to OD_600_ ∼0.3 and expression of the ECF41 σ factor was induced by adding IPTG. After 3 h of incubation, the cells were harvested, total RNA was prepared, and microarray analysis performed (see materials and methods for details). The mRNA level for *ecf*41_Rsp_ was ∼80-fold increased in cells overexpressing this protein. The only other more than twofold-induced gene was RSP_0606 (∼threefold), which encodes the associated carboxymuconolactone decarboxylase (data not shown). With this approach, we cannot discriminate between the expression level of the native and the ectopic copy of *ecf*41_Rsp_. Nevertheless, we propose that *ecf*41_Rsp_ and RSP_0606 form a bicistronic operon due to an overlap of the structural genes. Taken together, these results indicate that Ecf41_Rsp_ seems to control expression of only a single transcript that contains the *ecf*41_Rsp_ and RSP_0606 genes.

#### Analysis of ECF41-dependent target promoters in *B. licheniformis* and *R. sphaeroides*

Since ECF σ factors recognize alternative promoter sequences, we investigated if the identified ECF41 target genes *ydfG* and RSP_0606 are preceded by such a unique sequence motif. We therefore mapped the transcriptional start site by 5′RACE in RNA samples from cells overexpressing the ECF41 σ factor (*R. sphaeroides*) or samples taken throughout the growth cycle (*B. licheniformis*) (Fig. 4B). In both organisms, we identified a “G” residue as the transcriptional start site followed by a 22/23 bp untranslated region containing a suitable ribosome-binding site (Fig. 4A). Upstream of this start point, we identified a bipartite sequence motif with similarity to typical ECF-dependent promoter elements (Helmann 2002; Staroń et al. 2009): a –35 region identical in both organisms (TGTCACA) and a –10 region (TGTT or CGTC).

Next, we tested if this predicted target promoter responds to the corresponding ECF41 σ factor. Because of the lack of genetic tools for *B. licheniformis*, we heterologously expressed Ecf41_Bli_-FLAG from a xylose-inducible promoter in *B. subtilis*, an organism lacking an ECF41 σ factor, and measured the activity of the target promoter P*_ydfG_* trans-criptionally fused to *lacZ* by β-galactosidase assays (Fig. 4D). Without xylose, the resulting reporter strain TMB696 shows only low P*_ydfG_* activity, presumably due to weak basal expression of Ecf41_Bli_ from P*_xylA_* in complex LB medium. Addition of 0.5% xylose increased promoter activity ∼70-fold, indicating that Ecf41_Bli_ activates P*_ydfG_* upon its overexpression. Almost no β-galactosidase activity was detectable in the control strain TMB623, harboring only the empty expression vector, demonstrating that P*_ydfG_* from *B. licheniformis* is not recognized and activated by any σ factor of *B. subtilis* under the laboratory conditions used in these experiments.

To test for promoter recognition in *R. sphaeroides*, we expressed Ecf41_Rsp_ from an IPTG-inducible promoter and measured the activity of the target promoter P_RSP_0606_ by β-galactosidase assays. Without inducer present, strain TMR005 shows P_RSP_0606_ activity of about 25 Miller Units, which can be increased twofold by addition of IPTG (Fig. 4D). The high P_RSP_0606_ activity of TMR005 in the absence of IPTG is presumably due to background transcription from the leaky promoter of the expression plasmid. In comparison, the control strain YSD434, which carries the empty vector, shows only marginal promoter activity. These results from two independent organisms demonstrate that the promoter identified by 5′RACE (Fig. 4A) specifically responds to the overexpression of ECF41 σ factors.

#### Prediction of a general ECF41-dependent target promoter motif

After we identified an ECF41-dependent promoter in two organisms, we expected that this motif should also be present in the ECF41 loci of other organisms. To verify this, we extracted 250-bp regions upstream of the COE- as well as the ECF41-encoding genes and searched for overrepresented sequence motifs with similarity to the identified promoter, either manually or by using the MEME algorithm (Bailey and Elkan 1994). We identified the two motifs described above separated by a 16 ± 1 bp spacer within most of these regions and constructed a weblogo based on 285 sequences (Fig. 4C). The –10 region with the consensus “CGTC” is comparable to many typical ECF promoters, whereas the –35 consensus “TGTCACA” is specific for group ECF41. This bipartite promoter motif can be found upstream of both the COE- and ECF41-encoding genes, often preceding a potential operon consisting of these two genes (Tables 4 and S1). In about one-third of all ECF41 σ factors, the COE-encoding gene is located upstream of and in the same orientation than the ECF gene. Here, the COE gene usually carries the promoter motif while the ECF41 σ factor lacks it. In case of this predominant locus organization, both genes could form an operon transcribed from the COE promoter. If the ECF σ factor is located upstream of the COE gene, less than 30% of the ECF- and almost 50% of the COE-encoding genes harbor the promoter, in case of 20% both genes are preceded by the motif. About 17% of the ECF41 σ factors show opposite orientation relative to the COE gene, either >ECF><COE< or <ECF< >COE>. In the latter case, often both genes are preceded by the motif, whereas for the first combination usually only either the ECF- or COE-encoding gene shows the promoter. More than 30% of all ECF41 σ factors do not show the genomic context conservation. Of these “orphan” genes, only 30% are preceded by the ECF41-specific promoter.

Next, we used the derived position weight matrix graphically represented in Figure 4C to perform genome-wide searches for additional ECF41 target promoters in *R. sphaeroides* and *B. licheniformis*, using the algorithm virtual footprint (Münch et al. 2005) implemented in the Prodoric database (Münch et al. 2003). In both organisms, only a few potential ECF41 target promoters could be identified (Table 5), but none exactly matched the ECF41 consensus. Construction of transcriptional *lacZ*-fusions to three of these promoters from *B. licheniformis* (P*_nhaX_*, P*_uvrX_*, and P*_ypbE_*) and subsequent determination of β-galactosidase activity did not reveal any Ecf41_Bli_-dependent activation. Even expression of a highly active truncated version of Ecf41_Bli_ (see below) did not result in any promoter activation (data not shown). In addition, we performed a genome-wide analysis on the presence and conservation of the ECF41-dependent promoter motif upstream of orthologous genes in a wide range of ECF41-harboring organisms. While this in silico analysis has been successfully used to identify candidate promoters and core regulons for other regulators including ECF σ factors (Dufour et al. 2008, 2010), it failed to reveal any potential conserved regulon members, with the exception of genes encoding the COEs or ECF41 σ factors (data not shown). Taken together, our collective data strongly suggest that the proteins of group ECF41 generally control expression of only a single target gene or operon, which includes the COE and the ECF41 σ factor, if cotranscribed. The COE-encoding genes therefore represent the only known and detectable targets of ECF41-dependent gene expression.

**Table 5:** Putative ECF41 target promoters in *B. licheniformis* and *R. sphaeroides*

Gene^1^	Promoter sequence^2^	5′UTR^3^	Putative function, homology
*B. licheniformis*			
BLi01248	TGTCACAAAAACATAAATAATAGATGTC	142	Hypothetical, putative membrane protein
Bli03073	TGTCACCCCTTCCTT-TTTCGAGCCGTC	109	Hypothetical, putative membrane protein
*hprP*	TGTCACGCTTGCTTTTATTTTTCTCGTC	163	Putative phosphatase
*mtrB*	TGTCACTTCAGCTGT-AAGGGGAACGTT	76	Transcription attenuation protein
***nhaX***	TGTCACGTTTAGGTG–CTTTTGTTGTT	199	Stress response protein
*pucR*	TGTCACAAATCCGCT–CATTTTTTGTT	39	Purine catabolism regulatory protein
*sat*	TGTCACAAGCGTTCTGCTGGCATCTGTC	97	Sulfate adenylyltransferase, dissimilatory-type
*spoIISB*	TGTCACAGAATTTGA-TTATCTCCTGTT	60	Stage II sporulation protein SB
***uvrX***	TGTCACCTTCTTTCC-AAAGAAGGTGTT	120	DNA-damage repair protein
*ybxF*	TGTCACTAAAAATTG-TCATCATATGTT	68	Firmicutes ribosomal L7Ae family protein
***ydfG***	TGTCACAAACTCCGT-TTCTCTCTTGTT	31	Putative carboxymuconolactone decarboxylase
*yfmE*	TGTCACGGCAATGAT-TGGGACGCCGTT	42	Heme ABC type transporter HtsABC, permease
*ykpA*	TGTCACAAAGAAAGTGGAAATAAGCGTT	108	ABC transporter, ATP-binding protein
***ypbE***	TGTCACGGCACATTTTTTGATCGATGTT	48	Unknown, LysM domain, cell wall degradation
*yvdI*	TGTCACACTGCTCATTTCTTTCATTGTC	63	Maltose/maltodextrin ABC transporter
*R. sphaeroides*			
*gcvH*	TGTCACGTCCGGCG-GCTTCGGCCCCTC	151	Glycine cleavage system H protein,
*repA*	TGTCACCGTTTCG–CCCCAAGAACGTG	152	RepA partitioning protein/ATPase, ParA type
*rplL*	TGTCACCCACC–ATGTTGGACCCCATC	20	Ribosomal protein
**RSP–0606**	TGTCACAACCGC-CTTCCCTCGCCCGTC	32	Putative carboxymuconolactone decarboxylase

1 Genes highlighted in bold were tested for activation by the corresponding ECF41 σ factor.^2^Underlining indicates –35 (left) and –10 (right) regions, the spacing was adjusted indicated by dashes.^3^5′UTR, length of 5′-untranslated region (in nucleotides) between the postulated transcriptional start site and the AUG start codon.

#### Phenotypes linked to ECF41-dependent gene expression

The analysis of the function of ECF41-dependent target genes could provide some hints for the physiological role of this novel group of ECF σ factors. The only detectable target gene of the ECF41 σ factor in both *B. licheniformis* and *R. sphaeroides* encodes a carboxymuconolactone decarboxylase, which is usually involved in the degradation of aromatic compounds (Eulberg et al. 1998; Lorite et al. 1998). But besides this function, proteins annotated as carboxymuconolactone decarboxylases can also exhibit a role in the oxidative stress response (Hillas et al. 2000; Lessner and Ferry 2007). Additionally, a strain of *M. tuberculosis* lacking the ECF41 σ factor σ^J^ is slightly more sensitive to H_2_O_2_ (Hu et al. 2004). Based on these observations, we investigated a potential link between ECF41 σ factors and oxidative stress response. We performed serial dilution spot tests to compare the viability of an *R. sphaeroides* wild-type (YSD354) and RSP_0606-*ecf*41_Rsp_ deletion mutant (YSD331) or Ecf41_Rsp1–206_ overexpression strain (TMR003, expressing a highly active version of Ecf41_Rsp_, see below) as well as *B. licheniformis* wild-type (MW3) and Δ*ecf*41_Bli_ (TMBli006) or Δ*ydfG* (TMBli003) deletion strains, respectively. No significant differences were observed in the presence of the oxidative stress generating compounds H_2_O_2_, cumene hydroperoxide, *t*-butyl hydro-peroxide, or paraquat (data not shown).

We subsequently performed a phenotype microarray (PM) analysis. This high-throughput approach allows testing hundreds of different conditions in parallel in order to identify phenotypes associated with genetic alterations (Bochner 2003). Our PM analysis included 960 assays for carbon, nitrogen, phosphorus, and sulfur utilization, nutrient stimulation, pH and osmotic stress, as well as chemical sensitivity tests covering 240 different substances (see http://www.biolog.com/PM_Maps.html for details). We compared phenotypic differences between the *R. sphaeroides* wild-type (2.4.1) and RSP_0606-*ecf*41_Rsp_ deletion strain (YSD239) as well as a strain expressing the highly active variant of Ecf41_Rsp_ (TMR003; see below). Overall, only very few phenotypes can be linked to the expression or deletion of the ECF41 σ factor. Besides resistance to spectinomycin due to the resistance cassette, strain YSD239 showed only a positive phenotype against the sulfonamide anti-biotic sulfadiazine. As expected, strain TMR003 displayed relative resistance to aminoglycoside antibiotics (kanamycin, neomycin, geneticin, paromomycin) due to the resistance cassette of the overexpression plasmid. Surprisingly, gained phenotypes were observed for the carbon sources adonitol, d-mannitol, d-sorbitol, and glucose, suggesting a metabolic function of the ECF41 σ factors in utilization of additional carbon sources. But none of these additional phenotypes not due to the presence of a resistance cassette could be verified by serial dilution spot tests (data not shown). Taken together, the mutant and overexpression strains do not show any reproducible growth differences compared to the wild type under all the conditions tested. Therefore, we were not able to identify an ECF41-related phenotype.

### Signal transduction of ECF41 σ factors

The activity of ECF σ factors is normally regulated by a cognate anti-σ factor. The genes encoding these two proteins are usually located next to each other on the chromosome and cotranscribed (Helmann 2002; Butcher et al. 2008). As mentioned above, no obvious anti-σ factor is encoded in direct vicinity to the genes encoding the ECF41 σ factors. The results of our in silico analysis (Figs. 2 and 3) suggest that either the corresponding COEs or the C-terminal extension of the ECF41 proteins could be involved in the regulation of σ factor activity. We first tested if YdfG has any influence on the target promoter activation by Ecf41_Bli_. Therefore, we expressed Ecf41_Bli_ separately (TMB455) and simultaneously with YdfG (TMB456) in *B. subtilis* and measured the activity of P*_ydfG_* fused to *lacZ* by β-galactosidase assays, but did not observe any significant differences (data not shown). Therefore, we do not have any indication that the corresponding COEs are involved in ECF41-dependent signal transduction and focused our attention on the C-terminal extension. To study a possible function of this extension, we investigated the effect of C-terminal truncations of Ecf41_Bli_ on promoter activation and RNAP interaction.

#### Sequential deletion analysis of the C-terminal extension

**Figure 5:**
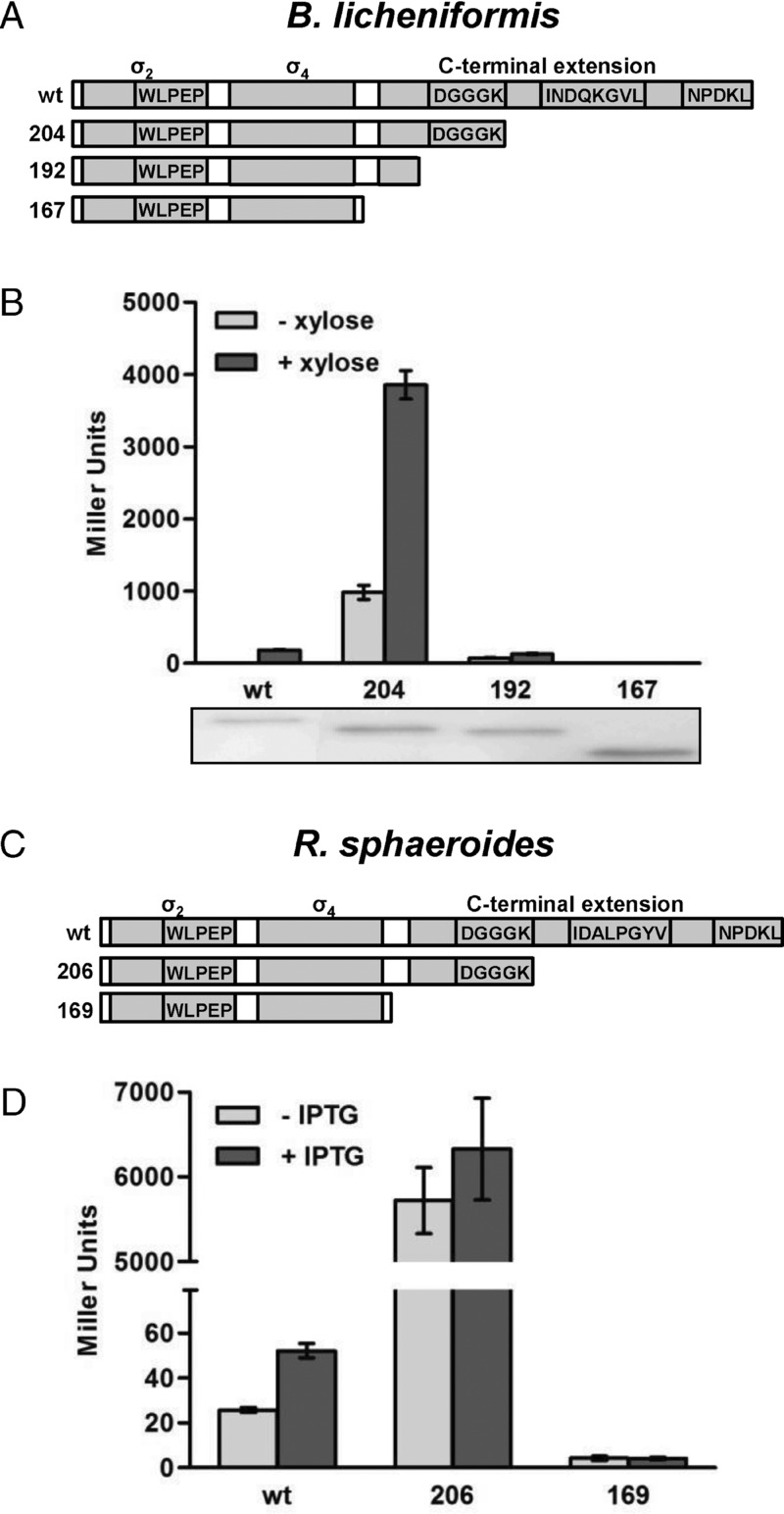
Effect of C-terminal truncations of ECF41 σ factors on target promoter activation. (A) Schematic representation of C-terminally truncated Ecf41_Bli_ proteins of *B. licheniformis*. The name of each variant is given at the beginning of each line and correlates with the length of the protein. The domains σ_2_ and σ_4_ as well as the C-terminal extension are displayed as gray boxes. The amino acid sequence of the highly conserved motives is shown. (B) β-galactosidase activities of *B. subtilis* strains overexpressing truncated Ecf41_Bli_-FLAG proteins. Strains TMB696 (wt), TMB742 (204), TMB741 (192), and TMB667 (167) were grown in LB medium to OD_600_ ∼0.4 and split into two samples. In one sample, protein expression was induced by addition of 0.5% xylose. The cells were harvested after 1 h and β-galactosidase activity was measured. Expression of the Ecf41_Bli_ variants was verified by Western Blot using a FLAG-tag-specific antibody and is shown below. (C) Schematic representation of C-terminally truncated Ecf41_Rsp_ proteins of *R. sphaeroides*. Details are shown as described for Figure 4. (D) β-galactosidase activities of *R. sphaeroides* strains overexpressing truncated Ecf41_Rsp_ proteins. Strains TMR005 (wt), TMR006 (169), and TMR007 (206) were grown in Sistrom's minimal medium to OD_600_ ∼0.4 and split into two samples. In one sample, expression of Ecf41_Rsp_ variants was induced by 100 μM IPTG. After 3 h, the cells were harvested and β-galactosidase assays were performed.

We constructed three C-terminally truncated alleles of *ecf*41_Bli_-FLAG and tested their ability to activate the target promoter P*_ydfG_*. The different mutant proteins were named according to their length (Fig. 5A). Truncation of the C-terminal part of the extension (204) resulted in a very active ECF σ factor leading to an ∼20-fold increase in P*_ydfG_* activity relative to the full-length protein. Surprisingly, further truncation (192) of Ecf41_Bli_ resulted in clearly decreased target promoter activation. Furthermore, expression of variant 167, which lacks the whole extension, completely lost the ability to activate the target promoter (Fig. 5B). This was unexpected, since regions σ_2_ and σ_4_ are usually sufficient for promoter recognition and activation by other ECF σ factors. This indicates that at least N-terminal parts of the extension are required for ECF41-dependent promoter activation, although partly truncations lead to a highly active protein. Expression of all of these Ecf41_Bli_-FLAG variants was verified by Western Blot (Fig. 5B).

To test if similar results can be observed for Ecf41_Rsp_, we tested the effects of C-terminal truncations on σ factor function (Fig. 5C and D). Expression of the C-terminal truncated Ecf41_Rsp_ (206) led to ∼120-fold higher P_RSP_0606_ activity than the full-length protein, whereas deletion of the whole extension (169) resulted in a total loss of promoter activation. Our collective data therefore strongly suggest that the C-terminal extension of group ECF41 proteins might represent a fused regulatory domain that is involved in controlling σ factor activity.

#### Interaction of Ecf41_Bli_ with RNA polymerase

**Figure 6:**
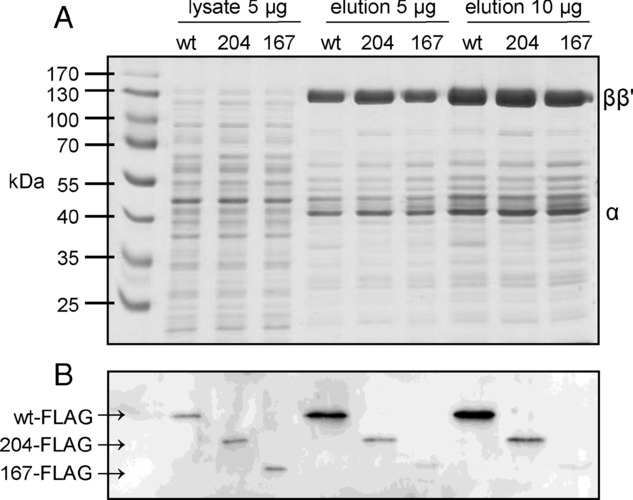
Interaction of Ecf41_Bli_ with RNA polymerase. (A) SDS-PAGE of Ni affinity-purified proteins from strains TMB1099 (wt), TMB1100 (204), and TMB1101 (167) carrying a His_6_-tag fused to the β′ subunit of the RNAP. The different truncated versions of Ecf41_Bli_-FLAG were overexpressed and the RNAP complex was purified (see Materials and Methods for details). A total of 5 μg of the cleared lysate and 5 and 10 μg of the 100 mM imidazole elution fraction were loaded. (B) Detection of copurified Ecf41_Bli_-FLAG and its variants by Western Blot analysis of a gel identical to the one in (A) using a FLAG-tag-specific antibody.

Bacterial σ factors form a complex with the RNAP core enzyme and recruit the resulting holoenzyme to the corresponding target promoters (Burgess and Anthony 2001). To demonstrate that Ecf41_Bli_ interacts with RNAP, we performed in vivo RNAP pull-down assays. Ecf41_Bli_-FLAG was expressed from a xylose-inducible promoter in a *B. subtilis* strain carrying a His_6_-tagged β′-subunit of RNAP. The His_6_-tag was used for rapid purification of RNAP holoenzyme (Anthony et al. 2000). The success of the purification was verified by the presence of bands on a Coomassie-stained SDS-PAGE gel corresponding to the ββ′ and α subunits (Fig. 6A). Western Blot analysis with a FLAG-tag-specific antibody shows that Ecf41_Bli_-FLAG copurifies with RNAP (Fig. 6B). The same protein is not detectable in the elution samples of cells lacking the His_6_-tag (data not shown) indicating that enrichment of Ecf41_Bli_-FLAG from *B. licheniformis* is due to interaction with RNAP.

Next, we analyzed if the observed effect of Ecf41_Bli_ truncations (Fig. 5A and B) on target promoter activation can be explained by their ability to interact with RNAP core enzyme. We repeated the RNAP pull-down assay quantitatively with the highly active (204), the inactive (167), and the wild-type version of Ecf41_Bli_-FLAG. All three proteins are expressed at comparable levels in soluble form as demonstrated by Western Blot analysis of the cleared lysate before purification, but show considerable different binding behavior to RNAP (Fig. 6B). Hardly any binding can be observed for the shortest Ecf41_Bli_ protein (167), which is consistent with its inability to activate the target promoter (Fig. 5B). In contrast, the protein with only partly truncated extension (204) co-purifies with RNAP to a much lesser extent than the full-length protein although promoter activation is significantly higher.

Taken together, C-terminal truncations of Ecf41_Bli_ significantly alter the binding behavior to RNAP, but this affinity does not completely reflect the observed σ factor activity of the corresponding allele. While loss of promoter activation for the complete deletion of the C-terminal extension can be—at least partially—explained by the significantly reduced ability of the shortest version of Ecf41_Bli_ (167) to interact with RNAP, additional factors must account for the strongly increased promoter activation of the partly truncated version (204), despite its weaker interaction with RNAP compared to the wild-type protein.

Taken together, our data demonstrate that the ECF41 σ factors seem to have a completely new way of signal trans-duction presumably not involving a second protein functioning as an anti-σ factor. Instead, the C-terminal extension, which is not present in other ECF σ factors, affects both the target promoter activation and binding to RNAP (Figs. 5 and 6). The mechanism of this mode of regulation remains to be investigated in subsequent studies.

## Discussion

A recent classification identified a large number of novel groups of ECF σ factors with unique features compared to “classical” ECF σ factors (Staroń et al. 2009), including group ECF41. This group shows a wide phylogenetic distribution with about 400 proteins from 10 different phyla (Tables and S1; Fig. 1). The genomic context of group ECF41 is highly conserved and distinct from other ECF groups. An obvious anti-σ factor is missing. Instead, a gene encoding a carboxymuconolactone decarboxylase, an oxidoreductase, or an epimerase (COE) is located directly up- or downstream of the ECF41 σ factor (Fig. 2). We did not observe any function of the COE proteins in signal transduction, but identified the neighboring genes encoding these proteins as the sole targets of ECF41 σ factors, both by in silico and comprehensive gene expression analyses. We identified a unique promoter signature (TGTCACA-n_16_-CGTC) upstream of these COE genes that is recognized by the corresponding ECF41 σ factor (Fig. 4).

The most important finding of our study concerns the regulatory role of the C-terminal extension of group ECF41, which is not present in any other group of ECF σ factors (Staroń et al. 2009). Based on our data, ECF41-dependent signal transduction does not seem to involve a second protein that functions as an anti-σ factor. Instead, our data clearly demonstrate the importance of the C-terminal extension for both target promoter activation and affinity to RNAP (Figs. 5 and 6). Moreover, our sequential deletion analysis indicates that the extension plays both a positive and negative role in ECF41-dependent gene regulation. A short N-terminal part of the extension directly following the region σ_4_ is absolutely required for σ factor activity, in contrast to other ECF σ factors described so far. But most of the C-terminal part of the extension clearly plays a negative regulatory role: even partial deletions result in a strongly increased activity of the target promoters in both organisms studied (Fig. 5), suggesting that this part of the extension functions as a fused inhibitory domain. To our knowledge, this is the first report of a regulatory domain being fused to the ECF σ factor. Based on our results, we propose that the group of ECF41 σ factors represents a novel mechanism of ECF-dependent signal transduction.

While our data clearly establish a regulatory role of the C-terminal extension for the activity of the ECF41 σ factor, the exact molecular mechanism will be the subject of further investigations. One possibility would be that the C-terminal extension functions as a sensory domain. In the absence of a suitable trigger, it could keep the σ factor domains inactive through intra- or intermolecular interactions. These interactions could prevent the binding of the ECF41 σ factor to RNAP or promoter DNA. The presence of an input signal could then result in a conformational change that releases the σ factor domains from the inhibitory grip of the extension, thereby initiating transcription of the COE genes. Such a mechanism involving intramolecular interactions has for example been described for the primary σ factor of *E. coli*, σ^70^. While this σ factor does not need to be activated, binding of free σ^70^ to DNA is inhibited by region 1.1, presumably by interaction with the σ_4_ DNA-binding domain (Dombroski et al. 1993). A similar inhibitory role of N-terminal regions has also been shown for alternative σ factors such as *E. coli* σ^32^ and *B. subtilis* σ^K^ (Dombroski et al. 1993; Johnson and Dombroski 1997). Another attractive but completely hypothetical possibility is that ECF41 proteins exist as inactive dimers in the cell with the extension being the dimerization interface. Activation would involve conformational changes and dissociation, resulting in a monomeric form that is able to interact with RNAP and promoter DNA. If a similar mechanism involving intra- or intermolecular interactions also applies to the C-terminal region of ECF41 σ factors needs to be investigated.

Alternatively, though maybe less likely, a stimulus sensed by the C-terminal regulatory domain could also result in a conformational change that exposes a protease recognition site. After regulated cleavage, the truncated and thereby activated σ factor would then mediate transcription initiation. Alternative σ factors such as σ^K^ and σ^E^, which are involved in the sporulation process in *B. subtilis*, are known to be expressed as inactive precursors. Activation is achieved by regulated proteolytic processing of the N-terminus of these proteins (LaBell et al. 1987; Lu et al. 1990; Zhang et al. 1998). But in addition to its inhibitory function, the role of the extension of ECF41 proteins seems to be more complex. Partial deletion of the C-terminal extension results in high activity, but at least the N-terminal part of the extension is also required for transcription. Since a complete deletion of the C-terminal extension seems to decrease the affinity of the ECF41 σ factor to RNAP (Fig. 5), the N-terminal part of the extension could be involved in stabilizing the complex of RNAP and σ factor. So far, we do not know any inducing conditions for ECF41-dependent gene expression. Hence, we could not investigate the influence of the C-terminal extension in signal transduction under natural conditions. Expression of Ecf41_Bli_ is already sufficient to activate the target promoter, showing that the full-length protein is not completely unable to initiate transcription. But the resulting activity can be drastically increased by truncation of the ECF σ factor. Since these expression and promoter activity experiments were carried out heterologously in *B. subtilis*, which does not possess an ECF41 σ factor, it is unlikely that another protein is involved in the signal transduction.

Future studies will be necessary to unravel both the physiological role and the mechanistic details underlying ECF41-dependent signaling. But the data presented in this initial study clearly demonstrate the value of our ECF classification (Staroń et al. 2009) and can serve as blueprint for studying additional conserved and novel groups of ECF σ factors, with yet to be explored mechanisms of signal transduction and gene regulation.

## Supporting information

Disclaimer: Supplementary materials have been peer-reviewed but not copyedited.

**Figure S1**. Alignment of ECF41 proteins.

**Table S1**. Excel file containing the nonredundant dataset with 373 proteins extracted from the MiST2 database (Ulrich and Zhulin 2010) in October 2010.

**Table S2**. Oligonucleotides used in this study.
