# Supplementary material for: Extracytoplasmic function σ factors of the widely distributed group ECF41 contain a fused regulatory domain
Source: Microbiologyopen. 2012 Jun;1(2):194–213. doi: 10.1002/mbo3.22 (PMC3426412; doi:10.1002/mbo3.22)
Supplement: Supplementary file 3 [file mbo30001-0194-SD3.doc]

# Supplemental Material for:

**Extracytoplasmic Function σ Factors of the Widely Distributed Group ECF41 Contain a Fused Regulatory Domain**

Tina Wecke, Petra Halang, Anna Staroń, Yann S. Dufour, Timothy J. Donohue, and Thorsten Mascher

**Supplemental Figures**

**Figure S1 (genbank file)**. Alignment of ECF41 proteins. The alignment containing 373 ECF41 protein sequences was constructed using ClustalW .

# Supplemental Tables

**Table S1**. Excel file containing the non-redundant dataset with 373 proteins extracted from the MiST2 database in October 2010. The dataset was analyzed regarding the genomic context and occurrence of the ECF41-dependent promoter motif. Pfam domains of the COE proteins were identified using the SMART database . Abbreviations: ECF, gene encoding the ECF41 σ factor; COE, gene encoding a carboxymuconolactone decarboxylase, oxidoreductases or epimerase; CMD, gene encoding a carboxymuconolactone decarboxylase; Ox, gene encoding an oxidoreductase; Epi, gene encoding an epimerase; Hypo, gene encoding a hypothetical protein.

**Table S2**. Oligonucleotides used in this study

| Number and name | Sequence |
| --- | --- |
|  |  |
| Construction of promoter *lacZ* fusions^a^ |  |
| 712 (P*_ydfG_* fwd (EcoRI)) | AGTC**GAATTC**CTTGGAATCCGGAAGGCGAT |
| 713 (P*_ydfG_* rev (BamHI)) | AGCT**GGATCC**CATTCCTCTGTATCCCTCAG |
| 1130 (P*_nhaX_* fwd (EcoRI)) | AGTC**GAATTC**GCACACTGTGTACCAGCATG |
| 1131 (P*_nhaX_* rev (BamHI)) | AGTC**GGATCC**TTCCGTCAAACGCGACTATG |
| 1132 (P*_uvrX_* fwd (EcoRI)) | AGTC**GAATTC**AAATTCCGAACTGGAATGGTC |
| 1133 (P*_uvrX_* rev (EcoRI)) | AGTC**GGATCC**AGCTCCGCATATCAACGCAC |
| 1136 (P*_ypbE_* fwd (EcoRI)) | AGTC**GAATTC**GATTGAGCTTTGAACGGACAG |
| 1137 (P*_ypbE_* rev (BamHI)) | AGTC**GGATCC**TCATTTCAGCGCTGGCCTTC |
| 109 (RSP_0606-*ecf*41_Rsp_ operon fwd) | AGGCAAAGTAGAGACCGCGTCC |
| 219 (RSP_0606 promoter rev (XbaI)) | ATGT**TCTAGA**CGCTCTCTCCTTTTGCAACTGA |
| 210 (*lacZ* start codon (ScaI, XbaI)) | CT**AGTACT**GTA**TCTAGA**TGACCATGATTACGGATTCA |
| 200 (*lacZ* terminator rev) | CATTACGGATCTTTTCTTTCG |
|  |  |
| Mutagenesis/expression experiments^a b c^ |  |
| 1416 (3xFLAG-*ecf*41_Bli_ fwd (BamHI)) | AGTC**GGATCC***AAGGAGGTGAGGATCTATGGATTATAAGGATCATGATGGTGATTATAAGGATCATGATATCGACTACAAAGACGATGACGACAAG*GAATATTATCGACAATATCATTC |
| 1294 (*ecf*41_Bli_ rev (AatII)) | AGTC**GACGTC**TTATATTTTAATGTGCTTCAGTTTATC |
| 1471 (*ecf*41_Bli_ 270 rev (AatII)) | AGTC**GACGTC***TCA*ATTTTTGACGGAATCGCCTTC |
| 1470 (*ecf*41_Bli_ 234 rev (AatII)) | AGTC**GACGTC***TCA*AAAGCGGCCGGAAAAGCTTC |
| 1469 (*ecf*41_Bli_ 204 rev (AatII)) | AGTC**GACGTC***TCA*CACTTTTCCGCCGCCATCT |
| 1468 (*ecf*41_Bli_ 192 rev (AatII)) | AGTC**GACGTC***TCA*TTCAATCAATTTCTTGGAAAACTC |
| 1411 (*ecf*41_Bli_ 167 rev (AatII)) | AGTC**GACGTC***TCA*TTCTTCAACCGGCTGTGAA |
| 699 (*ecf*41_Bli_ fwd (PacI)) | TACG**TTAATTAA**TTTTAGGCAAAATATCTATGGG |
| 669 (*ecf*41_Bli_ rev (BamHI)) | GACT**GGATCC**TTATATTTTAATGTGCTTCAGTTTATC |
| 705 (*ecf*41_Bli_-*ydfG* rev (BamHI)) | AGCT**GGATCC**CGCTCAAATAAAGTGAAAGATAG |
| 1576 (*ecf*41_Bli_ 204 rev (BamHI)) | AGTC**GGATCC***TCA*CACTTTTCCGCCGCCATCT |
| 1881 (*ecf*41_Rsp_ fwd (NcoI)) | GTCA**CCATGG**CGCCTGACGTCTACCTGCA |
| 1603 (*ecf*41_Rsp_ rev (HindIII)) | GTCA**AAGCTT**TCAGTTGAGCCTGATACGGGTC |
| 1604 (*ecf*41_Rsp_ 169 rev (HindIII)) | GTCA**AAGCTT***TCA*CGCTGCCTCGCGCTCCACCT |
| 1605 (*ecf*41_Rsp_ 206 rev (HindIII)) | GTCA**AAGCTT***TCA*GACCTTGCCGCCACCGTCCGA |
| 185 (*ecf*41_Rsp_ fwd (BsrDI)) | GTA**GCAATG**CATGTCGCCTGACGTCTACCTGCAG |
| 186 (*ecf*41_Rsp_ rev (HindIII)) | GTA**AAGCTT**TCAGTTGAGCCTGATACGGGTCAGC |
|  |  |
| Deletion mutants^a d^ |  |
| 779 (*ecf*41_Bli_ clean up fwd (BamHI)) | AGTC**GGATCC**TATCCAGCCGATTGTCGTCA |
| 780 (*ecf*41_Bli_ clean up rev) | GGAGTTTGTGACAAAAAACGAGACGCTCCCCCATAGATATTTTGC |
| 781 (*ecf*41_Bli_ clean do fwd) | CGTTTTTTGTCACAAACTCC |
| 782 (*ecf*41_Bli_ clean do rev (EcoRI)) | AGTC**GAATTC**ACCTACTTTCACATTGAACAAG |
| 783 (*ydfG* clean up fwd (BamHI)) | AGTCGGATCCTGGCGCTTTCAGCTCAAATC |
| 784 (*ydfG* clean up rev) | GTCTATTCCTCCTTTAAGTGTT |
| 785 (*ydfG* clean do fwd) | AACACTTAAAGGAGGAATAGACTTGAAATCCCCCCAACACAG |
| 786 (*ydfG* clean do rev (EcoRI)) | AGTCGAATTCCCGTCGATCATCAGATCCGT |
| 109 (RSP_0606- *ecf*41_Rsp_ operon fwd) | AGGCAAAGTAGAGACCGCGTCC |
| 110 (RSP_0606- *ecf*41_Rsp_ operon rev) | ACGGGTTGGCACGCTGGATGAG |
| 125 (RSP_0606 rev-inverted) | GCGTTTGAAATGGTCGGTCATGC |
| 126 (RSP_ *ecf*41_Rsp_ fwd-inverted) | TGACCCGTATCAGGCTCAACTG |
|  |  |
| Northern Hybridisation^e^ |  |
| 688 (*ydfG*-fwd) | CTGAGGGATACAGAGGAATG |
| 761 (*ydfG*-probe-T7-rev) | **CTAATACGACTCACTATAGGGAGA**CGATGGCAATCCTGTTCCAG |
|  |  |
| 5’RACE |  |
| 679 (RACE PCR) | GATATGCGCGAATTCCTG |
| 689 (GSP1-*ydfG*) | CTTGCATCCTTCGTATGCATA |
| 690 (GSP2-*ydfG*) | CACATCCGTTCAGCTGTGA |
| 1414 (GSP1-RSP_0606) | AGCGTGCATCTGCAGACAGA |
| 1415 (GSP2-RSP_0606) | AGCTGGATCCCATCTTCACCAGATGCAGCA |

^a^Restriction sites are highlighted in bold.

^b^Sequence of FLAG-tag is shown in underlined italics.

^c^Inserted sequences (stop codons or Shine-Dalgarno sequences) are highlighted in italics.

^d^Linker sequences for joining reactions are underlined.

^e^Sequence representing the T7 promoter for *in vitro* transcription is shown in bold and underlined.

**Supplemental References**

Letunic, I., R. R. Copley, B. Pils, S. Pinkert, J. Schultz & P. Bork, (2006) SMART 5: domains in the context of genomes and networks. *Nucl Acids Res* **34**: D257-260.

Schultz, J., F. Milpetz, P. Bork & C. P. Ponting, (1998) SMART, a simple modular architecture research tool: identification of signaling domains. *Proc Natl Acad Sci U S A* **95**: 5857-5864.

Thompson, J. D., D. G. Higgins & T. J. Gibson, (1994) CLUSTAL W: improving the sensitivity of progressive multiple sequence alignment through sequence weighting, position-specific gap penalties and weight matrix choice. *Nucleic Acids Res* **22**: 4673-4680.

Ulrich, L. E. & I. B. Zhulin, (2010) The MiST2 database: a comprehensive genomics resource on microbial signal transduction. *Nucleic Acids Res* **38**: D401-407.
